# A Versatile Toolkit for Semi-Automated Production of Fluorescent Chemokines to Study CCR7 Expression and Functions

**DOI:** 10.3390/ijms22084158

**Published:** 2021-04-16

**Authors:** Marc Artinger, Christoph Matti, Oliver J. Gerken, Christopher T. Veldkamp, Daniel F. Legler

**Affiliations:** 1 Biotechnology Institute Thurgau (BITg), University of Konstanz, Unterseestrasse 47, 8280 Kreuzlingen, Switzerland; marc.artinger@bitg.ch (M.A.); christoph.matti@bitg.ch (C.M.); oliver.gerken@bitg.ch (O.J.G.); 2 Graduate School for Cellular and Biomedical Sciences, University of Bern, 3012 Bern, Switzerland; 3 Department of Chemistry, University of Wisconsin-Whitewater, Whitewater WI 53190, USA; veldkamc@uww.edu; 4 Faculty of Biology, University of Konstanz, Universitätsstraße 10, 78464 Konstanz, Germany; 5 Theodor Kocher Institute, University of Bern, Freiestrasse 1, 3012 Bern, Switzerland

**Keywords:** fluorescent chemokine, CCL19, CCL21, CCR7, cell migration, leukocytes, flow cytometry, chemokine production

## Abstract

Chemokines guide leukocyte migration in different contexts, including homeostasis, immune surveillance and immunity. The chemokines CCL19 and CCL21 control lymphocyte and dendritic cell migration and homing to lymphoid organs. Thereby they orchestrate adaptive immunity in a chemokine receptor CCR7-dependent manner. Likewise, cancer cells that upregulate CCR7 expression are attracted by these chemokines and metastasize to lymphoid organs. In-depth investigation of CCR7 expression and chemokine-mediated signaling is pivotal to understand their role in health and disease. Appropriate fluorescent probes to track these events are increasingly in demand. Here, we present an approach to cost-effectively produce and fluorescently label CCL19 and CCL21 in a semi-automated process. We established a versatile protocol for the production of recombinant chemokines harboring a small C-terminal S6-tag for efficient and site-specific enzymatic labelling with an inorganic fluorescent dye of choice. We demonstrate that the fluorescently labeled chemokines CCL19-S6^Dy649P1^ and CCL21-S6^Dy649P1^ retain their full biological function as assessed by their abilities to mobilize intracellular calcium, to recruit β-arrestin to engaged receptors and to attract CCR7-expressing leukocytes. Moreover, we show that CCL19-S6^Dy649P1^ serves as powerful reagent to monitor CCR7 internalization by time-lapse confocal video microscopy and to stain CCR7-positive primary human and mouse T cell sub-populations.

## 1. Introduction

Chemokines, or chemotactic cytokines, are a family of small, secreted proteins that orchestrate cell migration [1,2]. Particularly, chemokines provide essential guidance cues for leukocytes and play a central role in the development and homeostasis of the immune system [3]. The human genome codes for at least 45 different chemokine proteins of 8–14 kDa. Chemokines are defined by their primary amino acid sequence and the presence of conserved cysteine residues, which form two structurally important disulfide bonds. They are grouped based on the position of the first two cysteines into CC (28 members), CXC (17 members), XC (2 members) and CX_3_C (1 member) chemokine ligands. Common structural features of chemokines further include a flexible, unstructured N-terminus, a central antiparallel three-stranded β-sheet and an overlaying C-terminal α-helix [1,2]. Functionally, they can be divided into inflammatory and homeostatic chemokines that are either locally induced upon injury and infection or that are produced constitutively at discrete sites.

Two members of the homeostatic CC motif chemokine family are CCL19 and CCL21, which share a 32% sequence identity [4]. Structurally, the most prominent difference between CCL19 and CCL21 is the prolonged C-terminus of CCL21 consisting of 37 positively charged amino acid enabling glycosaminoglycan binding and haptotactic gradient formation [5,6,7,8]. Both chemokines are predominantly secreted and presented by stromal cells of draining lymph nodes, the spleen and the lumen of high endothelial venules [9,10,11]. In addition, CCL19 is secreted by mature dendritic cells, whereas CCL21 is produced by lymphatic endothelial cells and medullary thymic epithelial cells [12,13,14,15]. CCL19 and CCL21 attract leukocyte subsets via their cognate chemokine receptor CCR7 [16,17]. The CCR7-CCL19/CCL21 axis is essential for the correct homing of various sub-populations and precursors of T cells and antigen-presenting cells to secondary lymphoid organs and the thymus [11]. Correspondingly, CCR7 is mainly expressed on the surface of semi-mature/mature dendritic cells (mDCs), thymocytes, naïve B and T cells, as well as central memory T cells [11,18]. Moreover, CCR7 expression is induced by various cancer types and promotes tumor cell migration and metastasis formation in lymphoid organs [19].

Classical chemokine receptors are serpentine, seven transmembrane domain receptors that couple to heterotrimeric G-proteins for signal transduction to control cell migration [2,20]. By contrast, atypical chemokine receptors are unable to initiate classical G-protein-dependent signaling pathways, but efficiently scavenge chemokines, and thereby are critical in the generation and maintenance of chemokine gradients [21]. Signal transduction of the classical chemokine receptor CCR7 is initiated upon binding of one of its two cognate chemokine ligands [8]. CCR7 triggering subsequently results in the activation of the heterotrimeric G-protein of the G_i_-subfamily resulting in the dissociation of the α-subunit from the ßγ-subunits of the G-protein; followed by the activation of phospholipase C and the production of second-messenger molecules, including intracellular Ca^2+^ and inositol phosphates [22,23,24]. In addition, CCR7 is phosphorylated on serine/threonine residues by either GPCR kinases or second-messenger-dependent protein kinases preferentially by CCL19 engagement, which is referred to as ligand biased signaling [8], leading to the recruitment of β-arrestins and subsequent receptor internalization and/or desensitization [8,22,25,26,27].

Rigorous analysis of expression patterns of chemokine receptors is frequently hampered by the lack of high-quality antibodies for detection. Critically, anti-chemokine receptor antibodies are commonly raised against linear peptides of extracellular receptor domains. However, chemokine receptors, including CCR7, frequently undergo cell-type and context specific post-translational modifications [28,29] and/or receptor oligomerization [30] that may mask epitope recognition. Hence, it regularly remains unclear whether an anti-chemokine receptor antibody evenly recognizes the different posttranslational modified forms of the receptor on the surface of individual cells, not to mention different cell sub-populations or cell types. Fluorescently labeled chemokines serve as excellent tools to overcome such restrictions and limitations that antibodies have. Notably, commercially available, but quite expensive fluorescently labeled CCL2 has effectively been used to characterize CCR2-positive leukocyte subsets [31] and to identify new cell types expressing the atypical chemokine receptor ACKR2 [32]. Recently, we have successfully expressed and purified secreted CCL19 and CCL21 as fusion proteins with monomeric red fluorescent protein (mRFP) from a transfected mammalian cell system [33]. Purified CCL19-mRFP and CCL21-mRFP were fully functional and served to monitor CCR7 and ACKR4 functions [33]. Notably, CCL21-mRFP helped to identify stroma cell subsets in thymus cell suspensions that are able to capture and present the chemokine [15]. A handicap of such fluorescent fusion proteins is that the fluorescent protein is more than twice the size of the chemokine.

Herein, we have designed a strategy for the semi-automated production of recombinant chemokines, which are subsequently labeled with a fluorescent dye. Chemokines can be generated in decent amounts as recombinant proteins in *Escherichia coli* [34,35]. Learning from nature, we choose to covalently link a fluorophore to a recombinant chemokine in an enzymatic reaction. *Bacillus subtilis* expresses a specific Sfp synthase transferring 4′-phosphopantetheinyl groups from CoenzymeA (CoA) to a conserved serine residue of acyl or peptidyl carrier proteins (ACP or PCP), a process which can also be found in *E. coli* [36,37,38]. Such transferases are tolerant towards a wide range of molecules attached to CoA, including inorganic dyes [39]. Notably, ACP or PCP domains recognized by synthases can be minimized to an 11-12 amino acid short motif sufficient for enzymatic labelling [39,40]. The Sfp synthase was shown to have the highest affinity for a small, 12 amino acid sequence generated from recent YbbR-tags, referred to as the S6-tag consisting of the amino acid sequence GDSLSWLLRLLN [40]. Hence, we engineered the chemokines CCL19 and CCL21 as S6-tagged recombinant proteins, which can be purified on an ÄKTA automated FPLC and HPLC system, and that can easily be enzymatically labeled with a fluorophore of choice.

## 2. Results

### 2.1. Recombinant Production, Automated Chromatography Purification and Enzymatic Fluorescent Labelling of CCL19 and CCL21

**Figure 1:**
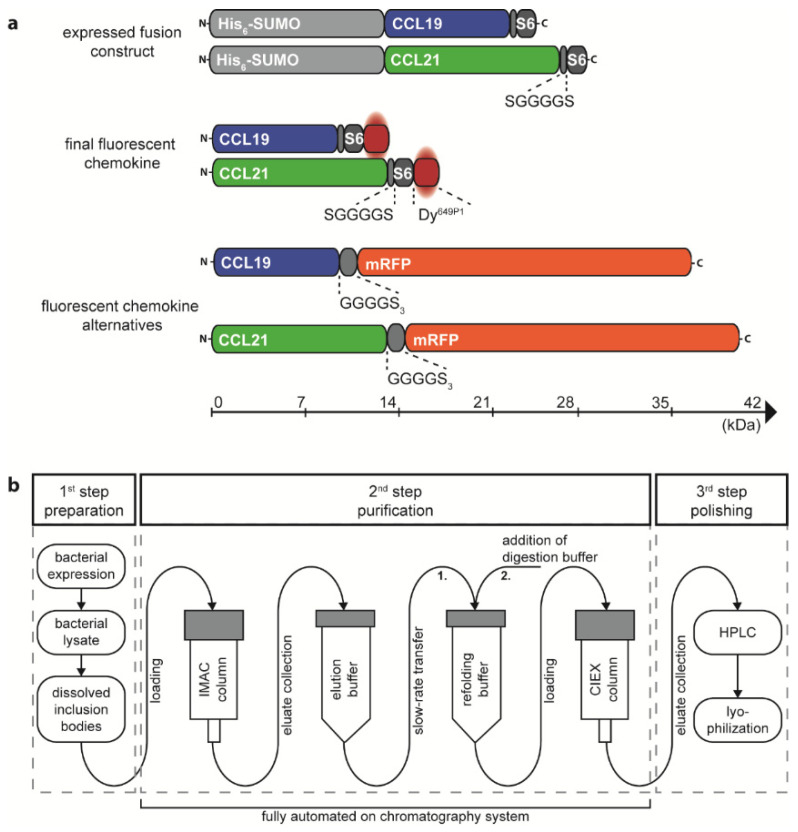
Design and production of the recombinant chemokines CCL19 and CCL21. (**a**) Schematic representation of the size matched expression constructs and the corresponding final fluorescently labeled chemokine proteins. A cleavable His_6_-SUMO-tag is fused to the N-terminus of mature human CCL19 and CCL21 followed by a short, flexible linker and the S6-tag for enzymatic labelling with the dye Dy^649P1^. Human CCL19-mRFP and CCL21-mRFP are included for size comparison. (**b**) Workflow overview of the chemokine production process. IMAC purification, chemokine refolding, and CIEX purification can be performed in an automated fashion on a chromatography system.

We have previously described a method to express and purify fluorescent CCL19 and CCL21 as fusion proteins with mRFP from the supernatant of transfected HEK293 cells, suitable to monitor chemokine receptor functions [33] and to identify stroma cell subsets in the thymus able to capture and present the chemokine [15]. A disadvantage of such an approach is that the fluorescent protein is more than twice the size of the chemokine itself and that it requests large quantities of transfected mammalian cells to produce the fluorescent chemokine fusion proteins. We therefore thought to establish an efficient and cost-effective method to produce and site-specifically label recombinant CCL19 and CCL21 in a semi-automated manner. Critically, random labelling or N-terminal modification of chemokines was not considered because such modifications are known to affect receptor binding and to frequently result in a loss of function [41,42]. To produce chemokines with a native N-terminus, we have adopted the protein His_6_-SUMO (also known as His_6_-SMT3) as a fusion protein (Figure 1a) to take advantage of the SUMO-specific protease Ulp1 (also known as SUMO protease) which cleaves the amide linking between the His_6_-SUMO-tag and the native chemokine [29,33,34,43]. At the chemokine’s C-terminus we included a short flexible linker (single letter amino acid sequence SGGGGS) followed by the S6-tag (single letter amino acid sequence GDSLSWLLRLLN) for enzymatic fluorescent labelling using the Sfp synthase and an inorganic dye of choice [40] (Figure 1a).

**Figure 2:**
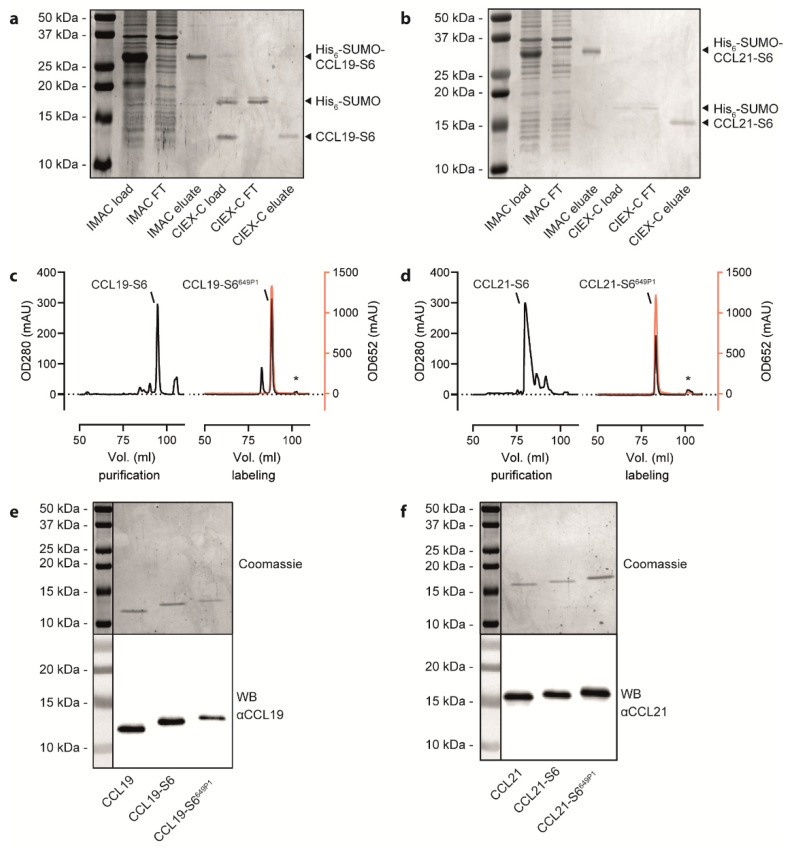
Purification and fluorescent labelling of human CCL19 and CCL21. (**a**,**b**) Representative Coomassie-stained SDS-PAGE gels with samples derived from the bacterial cell lysate before loading on the IMAC column, the IMAC flow through (FT), the IMAC eluate, the CIEX-C load after cleaving the His_6_-SUMO-tag, the CIEX-C flow through (FT) and CIEX-C eluate for the CCL19-S6 (**a**) and CCL21-S6 (**b**) purification steps illustrated in Figure 1b. (**c**,**d**) Representative HPLC chromatograms displaying the volume of gradient elution of CCL19-S6 (**c**) or CCL21-S6 (**d**) from a C-18 column used to separate correctly folded from misfolded chemokines (left panels) and to separate the fluorescently labeled chemokine from unlabeled chemokine, free substrate and the Sfp-CoA-Dy^649P1^ (marked with *). Protein concentration was determined by measuring its absorbance at 280 nm (OD280); fluorescence was measured at 652 nm (OD652) and illustrated in red. (**e**,**f**). Representative Coomassie-stained SDS-PAGE gels (upper panels) and corresponding Western blots (WB, lower panels) of the same samples of native, S6-tagged and S6-tagged, as well as fluorescently labeled CCL19 (**e**) and CCL21 (**f**).

The corresponding plasmids, pET-His_6_-SUMO-hCCL19-S6 and pET-His_6_-SUMO-hCCL21-S6, were expressed in the *E. coli* BL21(DE3) strain. The S6-tagged human chemokines can be purified from inclusion bodies and refolded following a detailed published protocol [34] and as described in the material and method section. We extended the standard chemokine purification and refolding procedure by introducing a 3-step purification process on an automated ÄKTA System, which allows easy manufacture upscaling associated with minimal manual process handling time (illustrated in Figure 1b). In a first step, bacterial inclusion bodies comprising the His_6_-SUMO-chemokine-S6 protein were dissolved and prepared for purification. In a second automated step, the sample was loaded on an immobilized metal (Ni^2+^) affinity chromatography (IMAC) column to separate the tagged chemokine from residual bacterial proteins (Figure 2a,b). The His_6_-SUMO-chemokine-S6 protein was automatically eluted and slowly transferred to a new reaction tube comprising refolding buffer, facilitating the refolding of the chemokine by infinite dilution. After refolding, digestion buffer was applied containing the Ulp1 protease specifically cleaving off the His_6_-SUMO protein yielding in a chemokine-S6 protein possessing the native, mature chemokine’s N-terminus (Figure 2a,b). The His_6_-SUMO protein was subsequently separated and removed from the chemokine by cation ion exchange chromatography (CIEX-C) within the ÄKTA automated purification process (Figure 2a,b). In a third polishing step, misfolded proteins were removed by reverse-phase HPLC, whereas the correctly folded chemokine was collected (Figure 2c,d), aliquoted and eventually lyophilized for storage.

Freshly prepared or reconstituted, recombinant human CCL19-S6 and CCL21-S6 were site-specific fluorescently labeled using the phosphopantetheinyl-transferase Sfp, which transfers the fluorescent dye (here Dy^649P1^) conjugated phosphopantetheine moiety of CoA to the first serine residue of the S6-tag. An additional reverse-phase HPLC purification step served to separate the fluorescently labeled CCL19-S6^649P1^ or CCL21-S6^649P1^, respectively, from residual substrate bound to the Sfp transferase (marked with asterisks in Figure 2c,d). Figure 2c,d illustrate the high efficiency of chemokine labeling using this strategy. SDS-PAGE followed by Coomassie-staining or Western blotting revealed the correct size of the recombinant chemokine variants (Figure 2e,f) that match the calculated molecular weights of native CCL19 (8.80 kDa), CCL19-S6 (10.50 kDa), CCL19-S6^649P1^ (12.58 kDa), as well as of native CCL21 (12.25 kDa), CCL21-S6 (13.95 kDa), and CCL21-S6^649P1^ (16.03 kDa).

### 2.2. Fluorescent CCL19-S6^649P1^ and CCL21-S6^649P1^ Are Biologically Active and Efficiently Elicit Early CCR7 Signal Transduction Pathways

**Figure 3:**
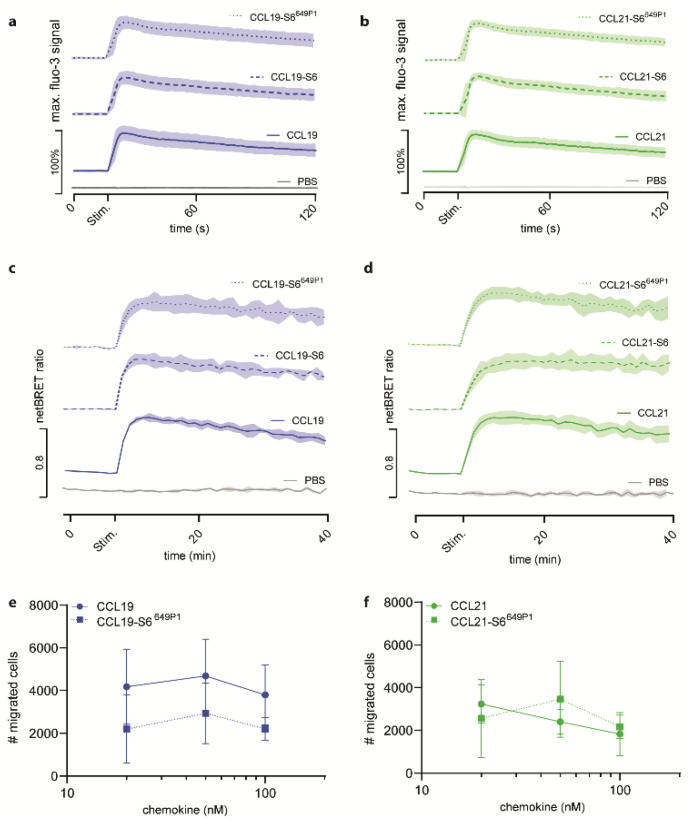
Fluorescent CCL19-S6^649P1^ and CCL21-S6^649P1^ efficiently elicit CCR7-mediated signaling and migration. (**a**,**b**) Pre-B 300-19 cells stably expressing CCR7 were stimulated with 50 nM of the indicated CCL19 (**a**) and CCL21 (**b**) chemokine variants and changes in intracellular calcium levels were recorded by flow cytometry over time. The time point of stimulation (Stim.) is indicated. Stimulation with PBS, the chemokine solvent, served as negative control. Mean values ± SD of three independent experiments are shown. (**c**,**d**) HeLa cells transiently transfected with CCR7-EGFP and β-arrestin2-Nluc were stimulated with 500 nM of CCL19, CCL19-S6, or CCL19-S6^649P1^ (**c**), and 1.5 µM CCL21, CCL21-S6, or CCL21-S6^649P1^ (**d**), respectively, and chemokine-mediated β-arrestin2 recruitment to CCR7 was determined by BRET. Mean values ± SD of three independent experiments are depicted. (**e**,**f**) Pre-B 300-19 cells stably expressing CCR7 were allowed to migrate towards graded concentrations of CCL19, CCL19-S6^649P1^ (**e**), CCL21, or CCL21-S6^649P1^ (**f**) for 3h in a Transwell migration assay. Migrated cells were counted by flow cytometry; random migration in the absence of chemokine was subtracted. Mean values ± SD of three independent experiments are shown.

To assess the biological activity of the fluorescently labeled chemokines, we investigated typical chemokine-induced early receptor mediated signal transduction events. To this end, we first measured chemokine-induced transient elevations of intracellular Ca^2+^ concentrations. We stimulated 300-19 pre-B cells stably expressing CCR7 with 50 nM of either recombinant untagged CCL19, tagged CCL19-S6, or tagged and fluorescently labeled CCL19-S6^649P1^ (Figure 3a). All three variants of CCL19 showed equal [Ca^2+^]_i_ mobilization profiles, whereas addition of PBS did not mobilize [Ca^2+^]_i_. Similarly, untagged CCL21, CCL21-S6, and CCL21-S6^649P1^ elicited equivalent transient elevations in intracellular calcium levels in CCR7-expressing cells (Figure 3b).

Next, we assessed the ability of our recombinant chemokines to recruit β-arrestin2 to CCR7. As expected, untagged CCL19 efficiently recruited β-arrestin2 to the receptor in a BRET assay with HeLa cells transiently expressing β-arrestin2-Nluc and CCR7-EGFP (Figure 3c). Stimulation with CCL19-S6 or CCL19-S6^649P1^ also proficiently recruited β-arrestin2 to CCR7 (Figure 3c). Similarly, robust and comparable β-arrestin2 recruitment to CCR7 was measured in response to CCL21, CCL21-S6, and CCL21-S6^649P1^ (Figure 3d).

Moreover, we tested the activity of the fluorescent chemokines to recruit leukocytes in Transwell cell migration assays. For this, pre-B 300-19 cells stably expressing CCR7 were allowed to migrate towards graded concentrations of CCL19 or CCL19-S6^649P1^ for 3 h. Maximal migration was achieved by 50 nM of either CCL19 form (Figure 3e), which is in agreement with previous studies [16,23]. Likewise, CCR7-expressing cells also readily migrated in response to CCL21 and CCL21-S6^649P1^, although with a slightly altered, but not significantly different kinetic (Figure 3f).

Collectively, these data provide clear evidence that the new approach of generating fluorescently labeled chemokines results in biologically active CCL19-S6^649P1^ and CCL21-S6^649P1^ that efficiently elicit early CCR7 signaling pathways.

### 2.3. CCL19-S6^649P1^ Specifically Binds to and Interacts with CCR7 on Transfected Cells

**Figure 4:**
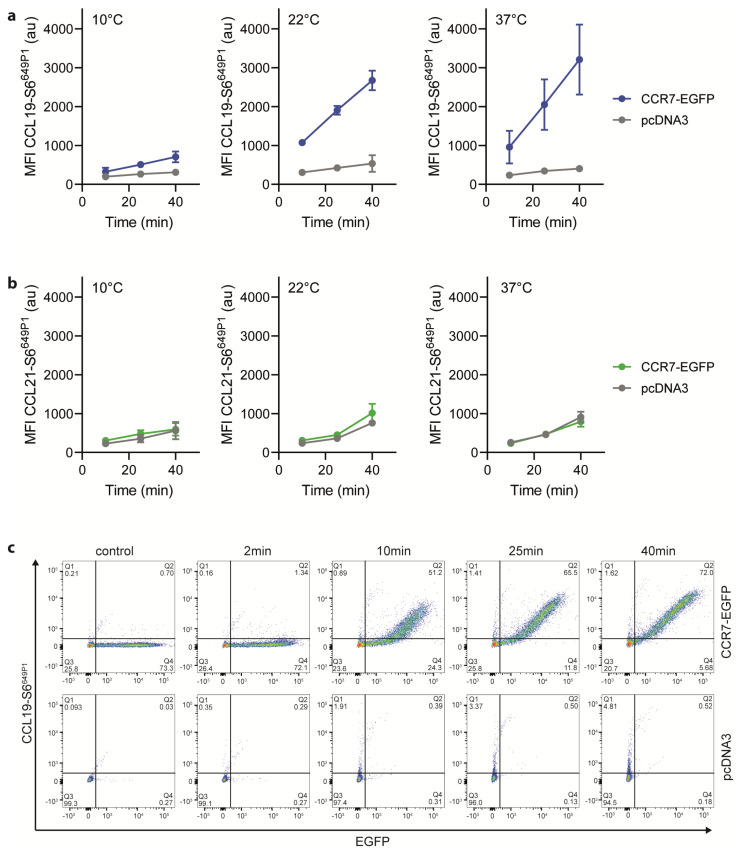
CCL19-S6^649P1^ readily and specifically binds to CCR7-expressing cells. (**a**,**b**) HeLa cells transiently transfected with CCR7-EGFP or its vector control (pcDNA3) were incubated at indicated temperature and time periods with 25 nM of CCL19-S6^649P1^ (**a**), or CCL21-S6^649P1^ (**b**). Cell associated, chemokine-derived mean fluorescence intensities (MFI) were recorded by flow cytometry. Mean values ± SD of three independent experiments are shown. (**c**) HeLa cells transiently transfected with CCR7-EGFP or its vector control (pcDNA3) were incubated at 22 °C with 25 nM of CCL19-S6^649P1^ for indicated time periods. Representative dot plots of one out of three experiments depicting CCL19-S6^649P1^ binding to CCR7-EGFP expressing and pcDNA3 vector control transfected cells.

To complement our functional activity results, we tested the binding abilities of the fluorescently labeled chemokines to CCR7-expressing cells. For this, we incubated HeLa cells transiently expressing CCR7-EGFP at various temperatures for different time periods with 25 nM of the fluorescently labeled chemokines. At 10 °C, CCL19-S6^649P1^ specifically bound to CCR7 transfected, but not to mock transfected cells (Figure 4a). Shifting the temperature to 22 °C, which still prevents receptor internalization, profoundly enhanced the specific binding of CCL19-S6^649P1^ to CCR7-expressing cells (Figure 4a). Cell associated, chemokine-derived fluorescence was highest upon incubation at 37 °C (Figure 4a), where CCL19-S6^649P1^ binds to CCR7 and subsequently promotes its co-internalization with the receptor [44]. Notably, CCL21-S6^649P1^ interacted with both CCR7-expressing and vector control transfected HeLa cells at all temperatures tested (Figure 4b). This is likely due to the increased binding ability of CCL21 to glycosaminoglycans and charged carbohydrates on the cell surface [5,6,8,45]. The enhanced GAG binding ability of CCL21 compared to CCL19 [46], suggests that CCL21-S6^649P1^ may be useful for visualizing the haptotactic CCL21 gradients rather than for labelling CCR7-expressing cells. Importantly, time-series experiments revealed that specific binding of CCL19-S6^649P1^ to CCR7-EGFP expressing HeLa cells, but not to vector control transfected cells, was easily recorded after 10 min and reached a plateau after 25 to 40 min of incubation at 22 °C (Figure 4c). Hence, CCL19-S6^649P1^ is an excellent fluorescent reagent to specifically mark CCR7-expressing cells.

### 2.4. CCL19-S6^649P1^ Is Internalized by CCR7-Expressing Cells

**Figure 5:**
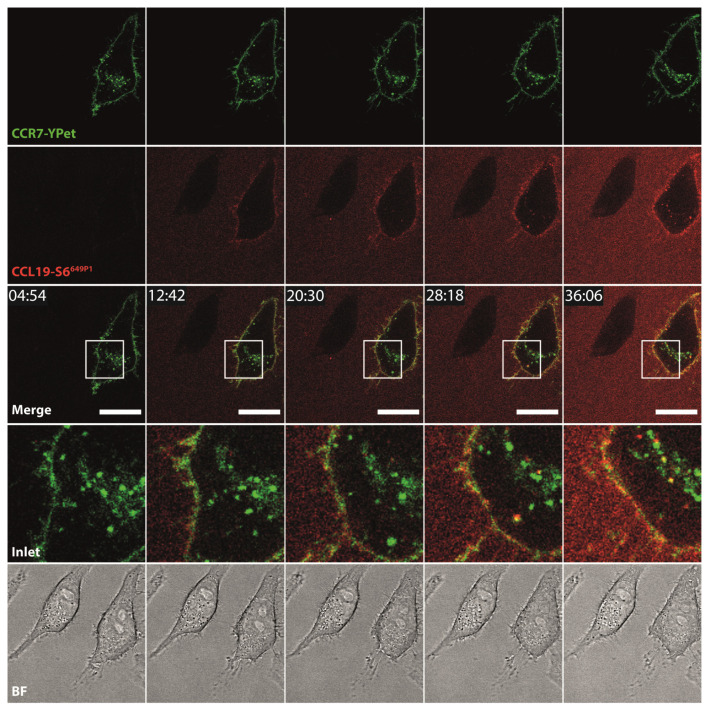
CCL19-S6^649P1^ binds to and is internalized specifically by CCR7-expressing cells. Time-lapse confocal video microscopy of HeLa cells transiently expressing CCR7-YPet stimulated at time point 10 min with 10 nM of CCL19-S6^649P1^ at 37 °C. Inlets (white rectangles) in merge images illustrate magnification, BF shows corresponding bright field images. Scale bar: 20 µm. Note that the neighboring cell lacking CCR7-YPet expression does not interact with CCL19-S6^649P1^. Images are representative for two independent experiments.

To further explore additional applications for the fluorescent chemokines, we incubated HeLa cells transiently expressing CCR7-YPet with fluorescently labeled CCL19-S6^649P1^ and followed receptor-mediated internalization of the chemokine by live-cell confocal video microscopy. We focused on CCL19 as only this ligand is efficiently internalized by CCR7 as reported previously [44,47]. Upon addition to the medium at 37 °C, CCL19-S6^649P1^ diffused within the medium and bound to CCR7-YPet expressed on the surface of transiently transfected HeLa cells. Subsequently, CCL19-S6^649P1^ was rapidly internalized by CCR7-YPet expressing cells where the chemokine co-localized with its receptor in intracellular vesicles (Figure 5, Supplementary Video 1). Critically, CCL19-S6^649P1^ did not bind to and was not internalized by neighboring, untransfected HeLa cells (Figure 5).

### 2.5. CCL19-S6^649P1^ Is a Versatile Tool to Stain CCR7-Expressing Primary Lymphocyte Subsets

**Figure 6:**
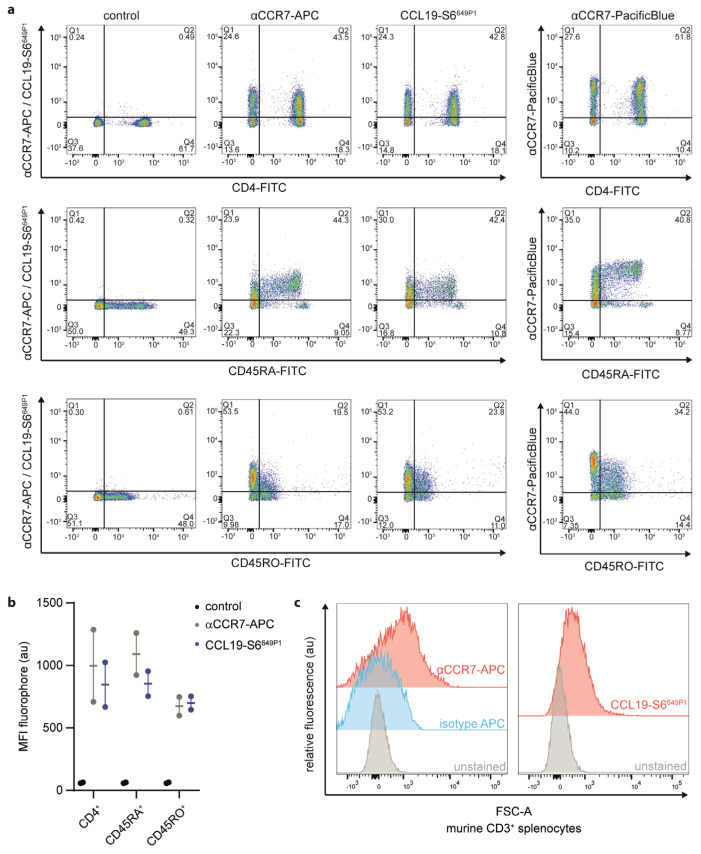
CCL19-S6^649P1^ is a versatile tool to stain CCR7-expressing primary human and mouse T cell sub-populations. Primary human peripheral blood CD3-sorted T cells (**a**,**b**) and mouse splenic T cells (**c**) were stained with 50 nM CCL19-S6^649P1^ for 20 min at 22 °C together with either anti-huCD4-FITC, anti-huCD45RA-FITC, anti-huCD45RO-FITC, anti-huCCR7-APC, anti-huCCR7-PacificBlue, anti-muCD3-PE, or anti-muCCR7-APC conjugated antibodies, respectively, and analyzed by flow cytometry. One (**a**) out of the two (**b**) human donors, or three (**c**) independent experiments with comparable results are shown.

To further explore CCL19-S6^649P1^ as tool to study endogenous CCR7 expression by primary lymphocytes, we isolated human peripheral blood mononuclear cells (PBMCs) from healthy donors and subsequently purified CD3^+^ T cells by MACS-sorting (Supplementary Figure S1a). We followed the optimized staining procedure established for CCR7 transfected cells to stain T cell sub-populations with our fluorescently labeled CCL19. We co-stained CD3-sorted T cells with CCL19-S6^649P1^ together with fluorescently labeled antibodies for CD4 (marking T helper cells), CD45RA (naïve T cells), CD45RO (memory T cells) or CCR7 (Figure 6a,b). Importantly, CCL19-S6^649P1^ marked the same T cell sub-populations as two distinct commercially available anti-CCR7 antibodies (Figure 6a). In fact, CCL19-S6^649P1^ marked about 40% of CD4^+^, ~40% of CD45RA^+^, and ~20% of CD45RO^+^ T cells derived from human peripheral blood. Notably, CCL19-S6^649P1^ binding does not interfere with the CCR7 antibody staining, and hence does not compete for the same binding site on the receptor (Supplementary Figure S1b). Finally, we also used our CCL19-S6^649P1^ to stain mouse CD3^+^ T cells isolated from the spleen. Indeed, CCL19-S6^649P1^ readily marked CCR7-expressing mouse T cells (Figure 6c).

In summary, we herein describe a method for the production, ÄKTA automated purification and fluorescent labelling of functional human CCL19-S6^649P1^ and CCL21-S6^649P1^. Moreover, we demonstrate that CCL19-S6^649P1^ serves as versatile tool to study CCR7 function and expression of primary human and mouse leukocyte sub-populations.

## 3. Discussion

In the present study, we have established a method for the cost-effective production, ÄKTA-automated FPLC and HPLC purification and fluorescent labelling of chemokines using the important lymph node homing chemokines CCL19 and CCL21 as a proof-of-concept. However, this approach is generally applicable to any other chemokine. To achieve this, we expressed the chemokines in a bacterial host system, which can easily be adapted for large-scale protein production. Importantly, chemokines are secreted proteins possessing a mature N-terminus that results from the removal of the signal peptide. The mature N-terminus is critical for its biological activity [41]. Hence, care must be taken when chemokines are produced as recombinant proteins because bacteria lack corresponding signal peptidases to properly process the chemokine’s signal peptide. Expressing chemokines without a signal peptide in bacteria is principally possible but introduces an N-terminal methionine to the mature protein, which often hampers chemokine functions. To circumvent these caveats, we fused a His_6_-SUMO-tag to the mature N-terminus of the chemokine. This facilitates on the one hand the efficient and straightforward affinity purification of the recombinant protein from bacterial inclusion bodies on Ni^2+^ columns. On the other hand, the His_6_-SUMO-tag can easily be removed by the SUMO-specific protease Ulp1, as Ulp1 recognizes the SUMO fold and specifically cleaves the amide bond between the His_6_-SUMO-tag’s C-terminus and the native, mature chemokine N-terminus [34,48]. For effective fluorescent labelling of the chemokine, we C-terminally introduced a short, six amino acid long flexible linker followed by a twelve amino acid long S6-tag. The S6-tag can be enzymatically and site-specifically labeled with the Sfp synthase [40] using fluorophore-conjugated CoA as substrate [39]. Most recombinant chemokines expressed in bacteria normally accumulate in high amounts in inclusion bodies and are unfolded. However, unfolded chemokines can be refolded by dialysis or infinite dilution [41,48]. An elegant approach is the on-column refolding of recombinant chemokines [49]. We further developed this approach to purify and refold the chemokines CCL19-S6 and CCL21-S6 on an automated ÄKTA chromatography system (Figure 1b). Our approach allows to cost-effectively produce decent amounts of fluorescent chemokines.

In general, various forms of commercially available or self-made fluorescent chemokines are sparsely available but have successfully been used to detect atypical and classical chemokine receptors on cell subsets, to sort chemokine receptor-positive cells, to study chemokine uptake or to investigate cell migration. For instance, CCL2-mCherry was used to identify the scavenging activity of CCR2 during monocyte migration [50]. An AlexaFluor-647-labeled CCL22 served to detect and characterize stromal cell populations expressing the atypical chemokine receptor ACKR2 upon intratracheal or intravenous application of the chemokine in mice [32]. A fluorescently labeled chimeric CXCL11_12 chemokine was exploited as unique tool to measure the scavenging activity of the atypical chemokine receptor ACKR3 by marginal zone B cells in vitro and in vivo [51,52]. Human CCL20s^Dy649P1^ and mouse CCL20y^AF647^ served to identify ACKR4 as the scavenger receptor for this chemokine in vitro and in vivo [43]. A site-specific fluorescently labeled CCL21 was used to investigate the heparinase-sensitive binding of the chemokine to CCR7 in receptor transfected HEK293 cells [35]. We have successfully used CCL19-mRFP and CCL21-mRFP to monitor CCR7 and ACKR4 functions in vitro [33]. In the present study, we used our newly produced and fluorescently labeled CCL19-S6^649P1^ to investigate early CCR7 signaling events and to monitor endocytosis of the chemokine/receptor complex. Moreover, we exploited CCL19-S6^649P1^ to label CCR7-expressing human and mouse T cell sub-populations in leucocyte cell suspensions. These fluorescently labeled chemokines seem also ideal tools to identify ACKR4-expressing cells and to study ACKR4 functions, such as chemokine scavenging, as well as chemokine gradient formation and maintenance. Overall, we present a cost-effective approach to produce decent amounts of fluorescently labeled chemokines as versatile tools to study and visualize chemokine receptor functions.

## 4. Materials and Methods

### 4.1. Reagents and Antibodies

Analytical-grade chemicals and reagents were purchased from Sigma (Sigma-Aldrich, Gillingham, UK) if not stated otherwise. Restriction enzymes for cloning were from Fermentas (ThermoFisher, Waltham, MA, USA). Antibodies from the following sources were used for Western blotting: mouse monoclonal anti-human CCL19 (R&D Systems, Minneapolis, MN, USA, #AB361), goat polyclonal anti-human CCL21 (R&D Systems, Minneapolis, MN, USA, #AF457), polyclonal rabbit anti-goat coupled to HRP (Dako Agilent, Santa Clara, CA, USA, #P0160), polyclonal goat anti-mouse-HRP (Jackson ImmunoResearch, West Grove, PA, USA, #115-035-003). For flow cytometry, the following antibodies were used: huCCR7-PacificBlue (Biolegend, San Diego, CA, USA, #353210), huCCR7-APC (R&D Systems, #FAB197A), huCD3-PacificBlue (Biolegend, San Diego, CA, USA, #300417), huCD4-FITC (Bio-Rad, Hercules, CA, USA, #MCA1267F), huCD45RA-FITC (Bio-Rad, Hercules, CA, USA, #MCA88F), huCD45RO-FITC (Bio-Rad, Hercules, CA, USA, #MCA461F), muCD3ε-PE (Biolegend, San Diego, CA, USA, #100307), muCCR7-APC (ThermoFisher, Waltham, MA, USA, #17-1971-82) and rat IgG2a kappa isotype control (ThermoFisher, Waltham, MA, USA, #17-4321-81). Dy^649P1^ (#649P1-03) was purchased from Dyomics GmbH (Jena, Germany) and conjugated to CoA as published previously [42].

### 4.2. Plasmids

The cloning of the plasmids pET-His_6_-SUMO-hCCL19 and pET-His_6_-SUMO-hCCL21 used for recombinant production of native, mature human CCL19 and CCL21 have been described previously [29,53]. The coding sequence for His_6_-SUMO-tagged chemokines were amplified by PCR using the forward primer primers 5′-CCC TCT AGA AAT AAT TTT GTT TAA CTT TAA GAA GGA GAT ATA CAT ATGG and the reverse primer 5′-CAG GTG CTC GAG TTA TTA GTT CAG CAG GCG CAG CAG CCA GCT CAG GCT ATC GCC GCT GCC GCC GCC GCC GCT ACT GCT GCG GCG CTT CAT CTTGG for CCL19 and 5′-CAGGTGCTC GAG TTA TTA GTT CAG CAG GCG CAG CAG CCA GCT CAG GCT ATC GCC GCT GCC GCC GCC GCC GCT TGG CCC TTT AGG GGT CTG TG for CCL21 was used, which introduced a SGGGGS-peptide linker and the S6-peptide tag (GDSLSWLLRLLN) to the C-terminal end of the chemokine. The amplified PCR fragments were re-cloned into the XhoI and XbaI restriction sites of pET-His_6_-SUMO revealing pET-His_6_-SUMO-hCCL19-S6 and pET-His_6_-SUMO-hCCL21-S6, respectively.

The plasmids pcDNA3-CCR7-HA [44] and pcDNA3-β-arrestin2i1-Nluc [53] have been reported previously. pcDNA3-CCR7-EGFP was cloned by amplifying human CCR7 from pcDNA3-CCR7-HA with primers inserting EcoRI and XhoI restriction sites (forward: 5′-GTA GCT CGA GTC CAC CTG GGG AGA AGG TGG TGG TGG TCT CG, reverse: 5′-GAA TTC CGT CAT GGA CCT GGG GAA ACC) and ligated into pcDNA3-ACKR4-EGFP [33]. For cloning of pcDNA3 CCR7-YPet, YPet was amplified from PL-452 N-YPet (Addgene #19172) with primers inserting XhoI and XbaI cutting sites and a (GGGGS)_3_-peptide linker (forward: 5′-GCA GAC TCG AGA GCG GAG GTG GCG GTT CTG GTG GTG GCG GTT CCG GCG GTG GCG GTA GCA TGG TGA GCA AAG GCG AAG AG, reverse: 5′-GCA GGT CTA GAT TAC TTA TAG AGC TCG TTC ATG CCC TC) and ligated into the corresponding cutting sites of pcDNA3-CCR7-EGFP.

### 4.3. Chemokine Production and Fluorescent Labelling

The production of different recombinant chemokines has been described previously [34,43,53]. Here, *E. coli* BL21(DE3) (Stratagene, La Jolla, CA, USA) were transformed with pET-His_6_-SUMO-hCCL19-S6 or pET-His_6_-SUMO-hCCL21-S6 and grown at 37 °C in modified LB medium (13.5 g/L peptone, 7 g/L yeast extract, 15 g/L glycerol, 2.5 g/L NaCl, 3.8 g/L sodium phosphate, 0.14 g/L magnesium sulfate, 0.01% antifoam B emulsion, pH 6.8) until they reached an OD_600_ of 0.6–0.8. Chemokine production was induced by adding 0.5 mM isopropyl-β-D-thiogalactopyranosid (IPTG) at 37 °C for 3–5h. After harvesting, bacterial pellets were re-suspended in buffer A (50 mM sodium phosphate, 300 mM NaCl, 10 mM imidazole, 2 mM tris(2-carboxyethyl)phosphine (TCEP), 1 mM phenylmethylsulfonyl fluoride (PMSF), pH 8.0) and lysed using a French Press (Constant Systems, Kennesaw, GA, USA) at 2.5 kbar. Inclusion bodies were collected by centrifugation at 25.000 g, 8 °C for 15 min and dissolved in buffer AD (50 mM sodium phosphate, 300 mM NaCl, 10 mM imidazole, 6M guanidine-HCl, 2 mM TCEP, pH 8.0) and passed through a 20 G syringe. After repeated centrifugation and filtration (0.45 µm), samples were loaded onto a HisTrapFF 5 ml column of an ÄKTA Pure 25M2 System equipped with an external sample pump (GE Healthcare, Chicago, IL, USA). Proteins were eluted using buffer BD (50 mM sodium phosphate, 300 mM NaCl, 10 mM imidazole, 6M guanidine-HCl, pH 4.5) and correct refolding was supported by infinite dilution into refolding buffer (50 mM Tris, 150 mM NaCl, 10 mM L-cysteine, 0.5 mM L-cystine, 1 mM EDTA, 1 mM PMSF, 10% glycerol, pH 8.5) before addition of digestion buffer (20 mM Tris, 1 mM EDTA, 1 mM PMSF, 10% glycerol, pH 8.0) containing 800 µg Ulp1 protease. After 5 h, chemokines were automatically loaded onto a HiTrap SP HP 5 ml column and eluted with cation ion exchange elution buffer (100 mM Tris, 2 M NaCl, pH 8.0). Fractions containing chemokines were pooled and further purified by reverse phase HPLC using a C18 column to separate correctly folded from misfolded chemokines. Recombinant chemokines were lyophilized and stored at −20 °C until use.

Recombinant S6-tagged CCL19 and CCL21 were fluorescently labeled using CoA-conjugated Dy^649P1^ (Dyomics, Jena, Germany; #649P1-03) and the phosphopantetheinyl transferase Sfp (New England Biolabs, Ipswich, MA, USA; #P9302S) in 50 mM HEPES, 10 mM MgCl_2_, 100 mM NaCl, 20% glycerol at 37 °C for 1h. Fluorescently labeled chemokines were re-purified by reverse phase HPLC to remove the unbound fluorescent substrate.

### 4.4. SDS-PAGE and Western Blot Analysis

Purified recombinant proteins or bacterial lysate samples were precipitated using ethanol or were directly mixed with 5× gel sample buffer, respectively. Proteins were denatured at 95 °C for 5 min and separated by SDS-PAGE. SDS-gels were stained with Instant Blue (Expedeon, Abcam, Cambridge, MA, USA) or semi-dry blotted onto nitrocellulose membranes (Whatman, GE Healthcare, Chicago, IL, USA) for Western blotting. Membranes were incubated with primary antibodies overnight at 4 °C followed by incubation with the corresponding secondary antibody at room temperature for 2h. Blots were developed using chemiluminescence using the Clarity Western ECL Substrate (Bio-Rad, Hercules, CA, USA) and analyzed on a ChemiDoc XRS Imaging System (Bio-Rad, Hercules, CA, USA).

### 4.5. Cell Lines and Transfection

Human HeLa cell cultures were maintained in DMEM (Pan Biotech, Aidenbach, Switzerland) supplemented with 10% fetal calf serum (FCS; Lonza, Basel, Switzerland) and 1% penicillin/streptomycin (P/S; Pan-Biotech, Aidenbach, Switzerland). Mouse 300-19 pre-B cells stably expressing CCR7 [29] were cultured in RPMI-1640 medium (Pan-Biotech, Aidenbach, Switzerland) containing 10% FCS, 1% P/S, 1% non-essential amino acids (Biowest, Riverside, MO, USA) and 0.1% β-mercaptoethanol (Gibco, ThermoFisher, Carlsbad, CA, USA). All cells were grown at 37 °C, 5% CO_2_ and 95% humidity.

Transient transfection of HeLa cells was performed using the Neon Transfection System (ThermoFisher, Waltham, MA, USA) according to the manufacturer’s protocol and the 100 µl transfection kit. In brief, 5 × 10^5^ cells were transfected with 10 µg of total plasmid DNA, pulsed twice for 35ms at 1.005V before seeding in pre-warmed DMEM supplemented with 20% FCS. Cells were used for subsequent experiments 36–48h post-transfection.

### 4.6. Isolation of Primary Human and Mouse Leukocytes

Human peripheral blood mononuclear cells (PBMCs) were isolated from fresh whole blood samples of voluntary healthy donors using BD Vacutainer CPT with an integrated Ficoll gradient (BD Biosciences, San Jose, CA, USA) according to the manufacturer’s protocol. Erythrocytes were removed by treating PBMCs with red blood cell lysis buffer (Biolegend, San Diego, CA, USA). Subsequently, CD3^+^ T cells were isolated using the Pan T cell Isolation Kit (Miltenyi Biotec, Bergisch Gladbach, Germany) and allowed to recover in RPMI-1640 medium (Pan-Biotech, Aidenbach, Switzerland) supplemented with 10% FCS (Lonza, Basel, Switzerland) overnight. Individual healthy donors gave written consent and blood donations were approved by the local ethics committee. Mouse splenocytes were isolated from the spleen of C57BL/6J mice kindly provided by the Animal Facility of the University of Konstanz. The organ collection was approved by the Review Board of Regierungspräsidium Freiburg in accordance with German Animal Protection Law (T-19/04TFA).

### 4.7. Chemokine-Mediated [Ca^2+^]_i_-Mobilization

To investigate chemokine-mediated changes in concentrations of intracellular free calcium, 300-19 cells expressing CCR7 were washed with pre-warmed calcium flux buffer (145 mM NaCl, 5 mM KCl, 1 mM MgCl_2_, 1 mM CaCl_2_, 1 mM sodium phosphate, 5 M HEPES, pH 7.5) and loaded with 4 mM Fluo-3 AM (ThermoFisher, Waltham, MA, USA) at 37 °C for 25 min. Cells were washed, adjusted to 3 × 10^6^ cells/mL and Fluo-3 fluorescence was recorded over time on a LSRII flow cytometer (BD Biosciences, San Jose, CA, USA). Cells were stimulated after 30 sec with 50 nM chemokine as indicated. Towards the end of an experiment, cells were treated with 1 µM ionomycin to determine maximal release of [Ca^2+^]_i_. Data were analyzed using the FACS Diva Software (BD Biosciences, San Jose, CA, USA).

### 4.8. Chemotaxis Assay

Two-dimensional cell migration assays were performed using 24-well Transwell plates (Corning, Corning, NY, USA) with 5 µm pore size. In brief, 1 × 10^5^ cells were seeded into the upper compartment allowing cell migration towards different concentrations of chemokines in the lower compartment. After 3h, migrated cells were collected and the cell number was determined by flow cytometry. The number of migrated cells was corrected for randomly migrated cells towards medium without chemokines.

### 4.9. Bioluminescence Resonance Energy Transfer (BRET)

HeLa cells were transiently co-transfected with pcDNA3-β-arrestin2i1-Nluc and pcDNA3-CCR7-EGFP or pcDNA3-CCR7-HA in a 1:1 ratio. After 36-48h, cells were washed with PBS supplemented with 5 mM glucose (PBS-G) and subsequently detached using Cell Dissociation Buffer (Gibco, ThermoFisher, Carlsbad, CA, USA) and collected in DMEM supplemented with 10% FCS. Cells were washed and resuspended in PBS-G and loaded with 5 µM of the luciferase substrate coelenterazine H (Biosynth, Staad, Switzerland, #C-7004) in a 96-well half-well plate (Perkin Elmer, Waltham, MA, USA). Luciferase luminescence (384–440 nm, 500 ms integration time) and EGFP fluorescence (505–590 nm, 500 ms integration time) were measured over time in a Tecan Spark 10M multiplate reader (Tecan, Männedorf, Switzerland) for 10 min before cells were stimulated with graded concentrations of chemokines. BRET ratio was calculated and netBRET was determined by baseline-correction for CCR7-HA transfected cells as described [43,53].

### 4.10. Chemokine Binding and Uptake Assay

HeLa cells were transiently transfected with CCR7-EGFP and seeded at a density of 1 × 10^5^ cells in 12-well plates. After 36–48h, cells were washed with PBS and pre-incubated at 10 °C, 22 °C or 37 °C for 15 min to equilibrate. Subsequently, cells were incubated with 25 nM of fluorescently labeled chemokines for various time points. Chemokine binding (at 10 °C or 22 °C) and chemokine uptake (at 37 °C) of CCR7-EGFP positive cells was determined after extensive washing on a BD LSRII flow cytometer (BD Biosciences, San Jose, CA, USA).

### 4.11. Chemokine Receptor Surface Staining

Human peripheral blood-derived CD3^+^ T cells and mouse splenocytes were washed with calcium flux buffer. In total, 1.5 × 10^5^ cells per assay point were incubated according to the manufacturer’s protocol at room temperature for 20 min with the corresponding antibody or with 50 nM fluorescently labeled chemokine. Cells were washed with calcium flux buffer and analyzed on a BD LSRII flow cytometer (BD Biosciences, San Jose, CA, USA).

### 4.12. Confocal Live Cell Imaging

HeLa cells were transiently transfected with CCR7-YPet and seeded into 35 mm µ-Dishes (Ibidi, Fitchburg, WI, USA). Adherent cells were washed and equilibrated at 37 °C with HEPES-buffered DMEM without phenol red (Gibco, ThermoFisher, Carlsbad, CA, USA) on a temperature-controlled TCS SP5 II (Leica, Wetzlar, Germany) for 10 min. Cells were stimulated with 10 nM of Dy^649P1^ fluorescently labeled CCL19-S6 and images were acquired using a 63× oil immersion objective. Acquired images were analyzed using FIJI [54].

## Data Availability

Datasets for this study are deposited on Zenodo and are publicly available under a Creative Commons Attribution 4.0 International license, doi:10.5281/zenodo.4636838.

## Supplementary Materials

The following are available online at https://www.mdpi.com/article/10.3390/ijms22084158/s1.
