# Supplementary material for: A Versatile Toolkit for Semi-Automated Production of Fluorescent Chemokines to Study CCR7 Expression and Functions
Source: Int J Mol Sci. 2021 Apr 16;22(8):4158. doi: 10.3390/ijms22084158 (PMC8072677; doi:10.3390/ijms22084158)
Supplement: Supplementary file 1 [file ijms-22-04158-s001.zip › Supplementary Figure S1.docx]

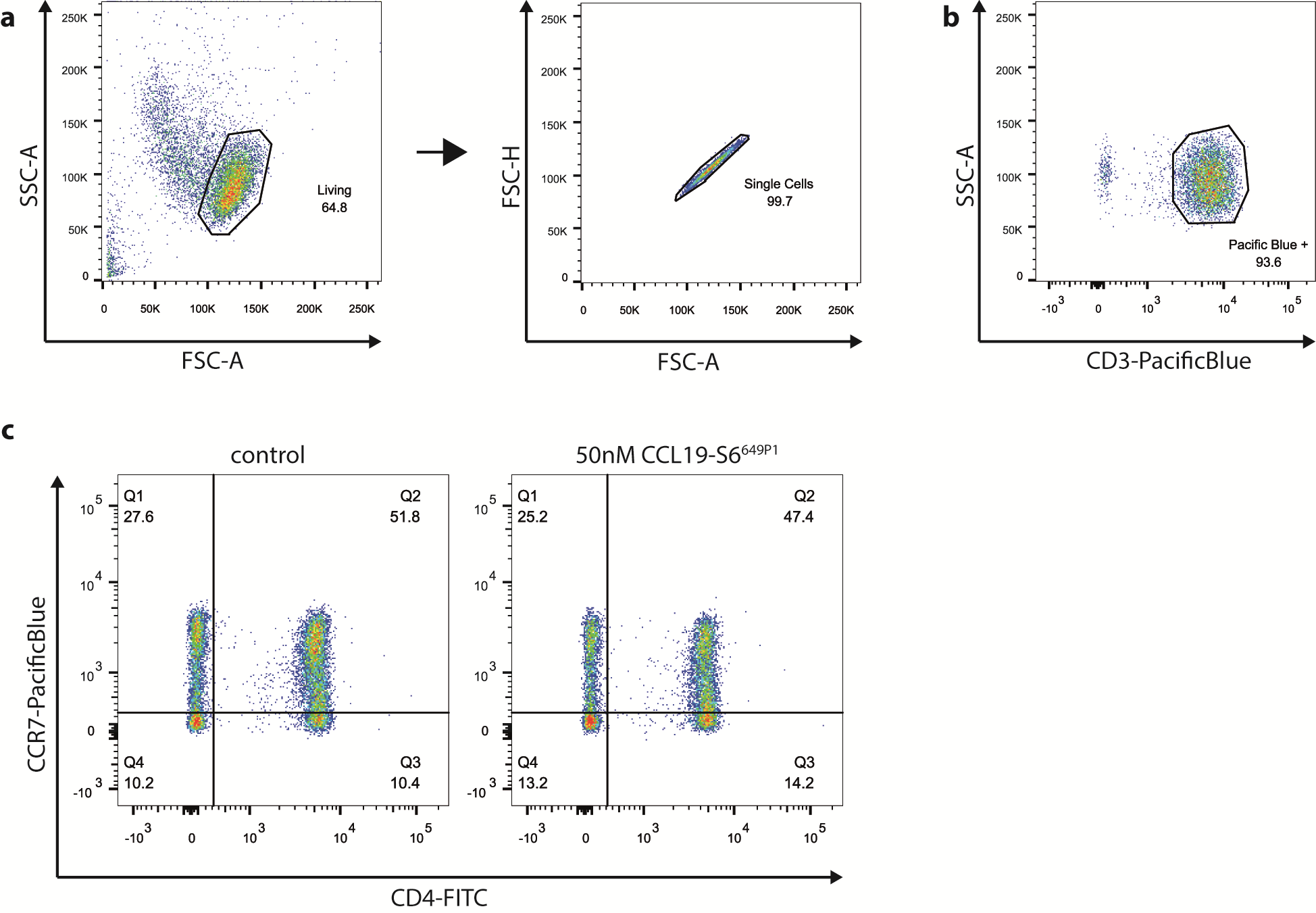


**Supplementary Figure S1.** Gating strategy and non-competitive labelling of CCR7 with CCL19-S6^649P1^ together with an anti-CCR7 antibody on primary human leukocytes. (**a**) flow cytometric gating strategy for the staining of singlet CD3^+^ T cells in human PBMCs. (**b**) flow cytometric assessment of the purity of CD3^+^ sorted human T cells. (**c**) Antibody co-staining of CCR7 and CD4 on CD3^+^ sorted human T cells incubated in the absence (left) or presence of 50nM CCL19-S6^649P1^ (right).
